# Exercise-Induced miR-210 Promotes Cardiomyocyte Proliferation and Survival and Mediates Exercise-Induced Cardiac Protection against Ischemia/Reperfusion Injury

**DOI:** 10.34133/research.0327

**Published:** 2024-02-26

**Authors:** Yihua Bei, Hongyun Wang, Yang Liu, Zhuhua Su, Xinpeng Li, Yujiao Zhu, Ziyi Zhang, Mingming Yin, Chen Chen, Lin Li, Meng Wei, Xiangmin Meng, Xuchun Liang, Zhenzhen Huang, Richard Yang Cao, Lei Wang, Guoping Li, Dragos Cretoiu, Junjie Xiao

**Affiliations:** ^1^ Institute of Geriatrics ( Shanghai University), Affiliated Nantong Hospital of Shanghai University (The Sixth People's Hospital of Nantong) and School of Life Science, Shanghai University, Nantong 226011, China.; ^2^ Joint International Research Laboratory of Biomaterials and Biotechnology in Organ Repair (Ministry of Education), Shanghai University, Shanghai 200444, China.; ^3^ Cardiac Regeneration and Ageing Lab, Institute of Cardiovascular Sciences, Shanghai Engineering Research Center of Organ Repair, School of Medicine, Shanghai University, Shanghai 200444, China.; ^4^ Department of Cardiology, Shanghai Tongji Hospital, Tongji University School of Medicine, Shanghai 200065, China.; ^5^ School of Environmental and Chemical Engineering, Shanghai University, Shanghai 200444, China.; ^6^ Cardiac Rehabilitation Program, Shanghai Xuhui Central Hospital/Zhongshan-Xuhui Hospital, Fudan University/Shanghai Clinical Research Center, Shanghai 200031, China.; ^7^ Department of Rehabilitation Medicine, Nanjing University of Chinese Medicine, Nanjing 210023, China.; ^8^ Cardiovascular Division of the Massachusetts General Hospital and Harvard Medical School, Boston, MA 02114, USA.; ^9^ Department of Medical Genetics, Carol Davila University of Medicine and Pharmacy, Bucharest 020031, Romania.; ^10^ Materno-Fetal Assistance Excellence Unit, Alessandrescu-Rusescu National Institute for Mother and Child Health, Bucharest 011062, Romania.

## Abstract

Exercise can stimulate physiological cardiac growth and provide cardioprotection effect in ischemia/reperfusion (I/R) injury. MiR-210 is regulated in the adaptation process induced by exercise; however, its impact on exercise-induced physiological cardiac growth and its contribution to exercise-driven cardioprotection remain unclear. We investigated the role and mechanism of miR-210 in exercise-induced physiological cardiac growth and explored whether miR-210 contributes to exercise-induced protection in alleviating I/R injury. Here, we first observed that regular swimming exercise can markedly increase miR-210 levels in the heart and blood samples of rats and mice. Circulating miR-210 levels were also elevated after a programmed cardiac rehabilitation in patients that were diagnosed of coronary heart diseases. In 8-week swimming model in wild-type (WT) and miR-210 knockout (KO) rats, we demonstrated that miR-210 was not integral for exercise-induced cardiac hypertrophy but it did influence cardiomyocyte proliferative activity. In neonatal rat cardiomyocytes, miR-210 promoted cell proliferation and suppressed apoptosis while not altering cell size. Additionally, miR-210 promoted cardiomyocyte proliferation and survival in human embryonic stem cell-derived cardiomyocytes (hESC-CMs) and AC16 cell line, indicating its functional roles in human cardiomyocytes. We further identified miR-210 target genes, cyclin-dependent kinase 10 (CDK10) and ephrin-A3 (EFNA3), that regulate cardiomyocyte proliferation and apoptosis. Finally, miR-210 KO and WT rats were subjected to swimming exercise followed by I/R injury. We demonstrated that miR-210 crucially contributed to exercise-driven cardioprotection against I/R injury. In summary, this study elucidates the role of miR-210, an exercise-responsive miRNA, in promoting the proliferative activity of cardiomyocytes during physiological cardiac growth. Furthermore, miR-210 plays an essential role in mediating the protective effects of exercise against cardiac I/R injury. Our findings suggest exercise as a potent nonpharmaceutical intervention for inducing miR-210, which can alleviate I/R injury and promote cardioprotection.

## Introduction

Cardiovascular mortality is predominantly attributed to coronary heart diseases including myocardial infarction [1]. Restoring coronary artery blood flow is the optimal strategy to limit infarct size, thereby preserving cardiac function and improving prognosis [2]. However, the cardiac ischemia/reperfusion (I/R) process can provoke additional myocardial damage after the primary ischemic injury [3–5]. Therefore, reducing I/R injury while treating myocardial infarction is key to prevent further myocardial damage and heart failure.

Exercise is regarded as a cost-effective strategy to boost cardiovascular health, and is often recommended as an important component of cardiac rehabilitation [6–8]. The cardiac protection conferred by exercise is attributed to several mechanisms [9], among which are molecular targets identified in physiological cardiac growth that might be potentially used to prevent myocardial injury [10]. Exercise stimulates physiological cardiac growth, characterized by both the cardiomyocyte enlargement in size, referred to as cardiomyocyte physiological hypertrophy, and a surge in cardiomyocyte proliferative activity [11]. Mechanistically, the insulin-like growth factor 1 (IGF1)/phosphatidylinositol 3-kinase (PI3K)/AKT pathway and the regulation of C/EBPβ transcription factor are pivotal to mediate physiological growth of the heart during exercise and can protect against pathological cardiac remodeling [12–14]. Notably, microRNA (miR-222), long noncoding RNA (CPhar), and circular RNA (circUtrn) have been revealed to regulate exercise-induced cardiac growth and exhibit cardioprotective effects [15–17]. With regard to cardiomyocytes, the enhanced proliferation and apoptosis-reducing effects are among the central mechanisms for exercise-induced myocardial protection [10]. Therefore, investigating the molecules that regulate exercise-induced cardiac growth is a promising way to develop new methods to prevent cardiac I/R injury.

miR-210, also known as miR-210-3p, has been known as a protective miRNA that protects against cardiac ischemic diseases [18]. MiR-210 is up-regulated upon hypoxia stress, while increasing miR-210 diminishes stem cell apoptosis and promotes cardiomyocyte survival [19–21]. MiR-210 can facilitate cardiac repair by augmenting cardiomyocyte survival as well as angiogenesis and mitochondrial metabolism in animals [22–24]. Interestingly, miR-210 has been revealed to be regulated by exercise [25,26]. This study investigated the function and the mechanism of miR-210 in exercise-induced physiological cardiac growth and explored its involvement in exercise-driven cardiac protection against I/R injury.

MiR-210 expressions were determined in the animal exercise models and in the blood samples from patients that were diagnosed of coronary heart diseases before and after cardiac rehabilitation. Utilizing miR-210 knockout (KO) rats, we established a swimming exercise model to investigate the effect of miR-210 in exercise-induced physiological cardiac growth. We then studied the functional roles of miR-210 and its potential downstream targets in primary neonatal rat cardiomyocytes (NRCMs), human AC16 cell line, and human embryonic stem cell-derived cardiomyocytes (hESC-CMs). Finally, the potential contribution of miR-210 to exercise-driven cardiac protection was evaluated in exercised miR-210 KO rats followed by cardiac I/R injury. Our research may offer a new mechanistic understanding for physiological cardiac adaptation during exercise and unravel the essential role of miR-210 in mediating exercise’s cardioprotection effect.

## Results

### Exercise induces miR-210 expression levels in both humans and experimental models

**Fig. 1.**
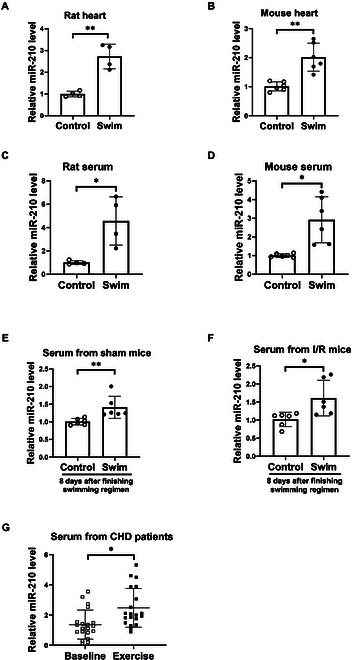
MiR-210 expression is increased in the heart and blood circulation upon exercise training. (A and B) qRT-PCR of miR-210 expression in the rat (A, *n* = 4) and mouse (B, *n* = 6) heart tissues after regular swimming exercise regimen. (C and D) qRT-PCR of miR-210 expression in the serum of rat (C, *n* = 4) and mouse (D, *n* = 6) after regular swimming exercise regimen. (E) qRT-PCR of miR-210 expression in the serum of mice at 8 days after finishing the swimming regimen (*n* = 6). Mice performed a 3-week swimming regimen followed by sham surgery. Serum samples were collected at 8 days after swimming exercise. (F) qRT-PCR of miR-210 expression in the serum of mice with cardiac ischemia/reperfusion (I/R) injury at 8 days after finishing the swimming regimen (*n* = 6). Mice performed a 3-week swimming regimen followed by cardiac I/R injury for 7 days. (G) qRT-PCR of miR-210 expression in human serum samples from patients with coronary heart diseases before and after 8 weeks of cardiac rehabilitation (*n* = 20). For statistical analysis, unpaired Student’s *t* test was performed for (A) to (D) and (F). Mann–Whitney *U* test was performed for (E). Paired Student’s *t* test was performed for (G) to compare the difference of human serum miR-210 expression levels before and after the cardiac rehabilitation program. Data are mean ± SD. *P < 0.05; **P < 0.01.

Murine models of swimming exercise were established, leading to physiological cardiac growth [15,27]. After a regularly programmed swimming regimen, miR-210 was markedly up-regulated in the murine heart tissues (Fig. 1A and B). Meanwhile, murine serum samples were collected after the swimming exercise regimen, showing that circulating miR-210 levels were also elevated upon exercise (Fig. 1C and D). To find out whether miR-210 was sustainably increased after the end of exercise, we also collected the serum samples from mice at 8 days after they had finished the swimming program where miR-210 was still increased in the swimming group (Fig. 1E). Interestingly, miR-210 could also be increased in the swimmed mice followed by cardiac I/R surgery (Fig. 1F). We further examined miR-210 levels in the human serums collected from patients diagnosed of coronary heart diseases after a programmed cardiac rehabilitation with targeted intensity [28]. Our results showed that miR-210 could also be induced in humans after aerobic exercise-based rehabilitation program (Fig. 1G). Consistently, these results indicate that regular exercise can induce miR-210 expression levels in both humans and experimental models.

### MiR-210 is not integral for cardiac hypertrophy but influences cardiomyocyte proliferative activity during cardiac adaptation upon exercise

**Fig. 2.**
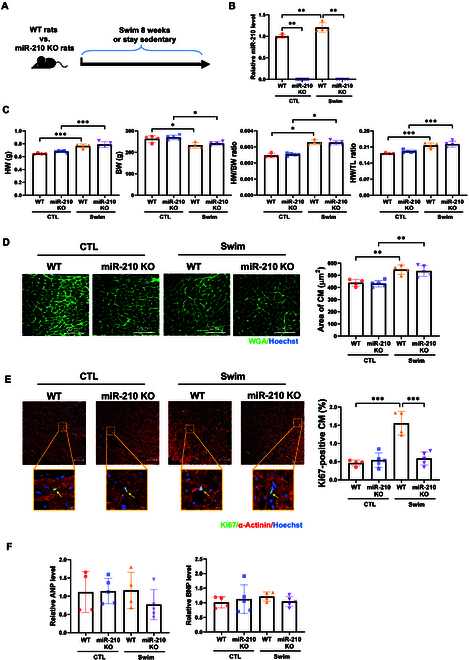
MiR-210 is involved in exercise-induced cardiomyocyte proliferative activity in vivo. (A) Schematic of rat swimming exercise model illustrating that adult wild-type (WT) versus miR-210 knockout (KO) rats took a swimming regimen or stayed sedentary for 8 weeks. (B) qRT-PCR of miR-210 expression in the heart (*n* = 4 to 5). (C) The heart weight (HW), body weight (BW), and HW relative to BW or tibia length (TL) were shown (*n* = 4 to 5). (D) Wheat germ agglutinin (WGA) staining to analyze cross-sectional myocardium area (*n* = 4 to 5). Scale bar, 50 μm. CM, cardiomyocyte. (E) Immunofluorescent staining for Ki67 and α-actinin for analyzing cardiomyocyte proliferative activity (*n* = 4 to 5). Scale bar, 100 μm. An enlarged area was shown below. Scale bar, 10 μm. (F) qRT-PCR of ANP and BNP expressions in the heart (*n* = 4 to 5). For statistical analysis, robust two-way ANOVA followed by post hoc pairwiseMedianTest was performed for (B) and (C) (HW/BW ratio). Two-way ANOVA test followed by Tukey post hoc test was performed for (C) (HW, BW, and HW/TL ratio) to (F). Data are mean ± SD. *P < 0.05; **P < 0.01; ***P < 0.001.

Cardiac growth as a result of exercise can encompass cardiac hypertrophy and increased cardiomyocyte proliferative activity. To clarify the function of miR-210 in this process, miR-210 KO rats underwent 8-week swimming exercise compared to WT controls (Fig. 2A). As demonstrated in Fig. 2B, following exercise, miR-210 was obviously up-regulated in WT rat hearts. Upon assessing heart weight (HW), cardiac hypertrophy, and cardiomyocyte proliferative activity in rats, we observed that swimming exercise markedly increased HW in both control and miR-210 suppressing rats; however, the HW increase after exercise did not show discernible differences between control and miR-210 suppressing rats (Fig. 2C). Interestingly, swimming exercise induced an equal increase in cardiomyocyte size in both WT and miR-210 KO rats (Fig. 2D), but the exercise-induced cardiomyocyte proliferative activity observed in WT rats was absent in miR-210 KO rats (Fig. 2E). Furthermore, the atrial natriuretic peptide (ANP) and brain natriuretic peptide (BNP) expression levels remained unaltered in WT or miR-210 KO exercised rat hearts, thus ruling out pathological hypertrophy in our experimental model (Fig. 2F). Collectively, these data demonstrate that miR-210 deficiency impedes exercise-induced cardiomyocyte proliferative activity but does not dampen exercise-induced cardiac hypertrophy.

### MiR-210 induces cardiomyocyte proliferation without affecting size

**Fig. 3.**
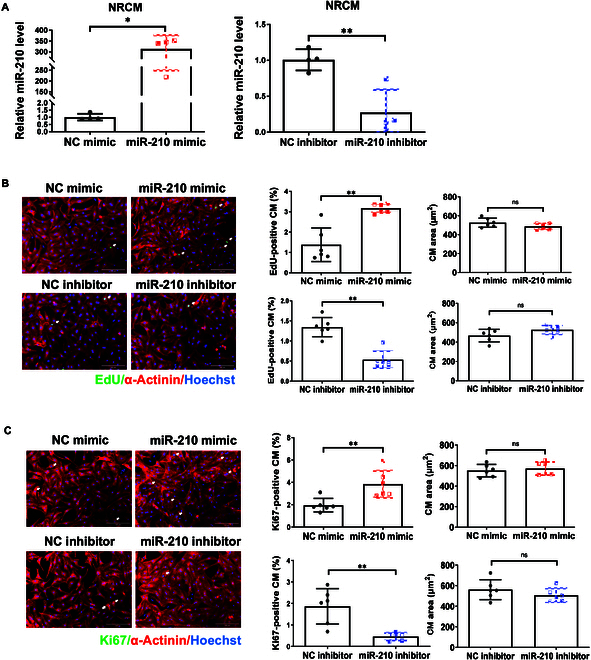
MiR-210 enhances proliferation but not cell size of cardiomyocytes in vitro. (A) qRT-PCR of miR-210 expression in neonate cardiomyocytes with transfection of miR-210 mimic or inhibitor (*n* = 4). NRCM, neonatal rat cardiomyocytes; NC, negative controls. (B and C) EdU/α-actinin (B, *n* = 6) or Ki67/α-actinin (C, *n* = 6) immunofluorescent stainings in NRCM with miR-210 overexpression or inhibition. Scale bar, 100 μm. For statistical analysis, Mann–Whitney *U* test was performed for (A) (miR-210 expression in NRCM transfected with miR-210 mimic) and (B) (EdU-positive CM in NRCM transfected with miR-210 inhibitor). Data in other figures were analyzed by unpaired Student’s *t* test. Data are mean ± SD. *P < 0.05; **P < 0.01; ns, not significant.

Following the in vivo study on miR-210’s function in the exercised model, we sought to further determine its influence on cardiomyocyte size and proliferation in vitro. Primary neonate cardiomyocytes were effectively transfected with mimic/inhibitor targeting miR-210 (Fig. 3A). Upon performing immunofluorescent staining for α-actinin with 5-ethynyl-2’-deoxyuridine (EdU), we found that overexpressing miR-210 increased EdU-positive NRCM (%), while inhibiting miR-210 reduced this; meanwhile, miR-210 was not shown to regulate cardiomyocyte size (Fig. 3B). Consistent with this, Ki67-positive cardiomyocytes showed that miR-210 promoted proliferation but did not alter cell size of NRCM (Fig. 3C). Using AC16 cardiomyocyte cell line, we observed similar effects of miR-210 in regulating proliferation (Fig. S1A to C). These in vitro functional studies demonstrate that miR-210 stimulates cardiomyocyte proliferation without influencing cardiomyocyte size.

### MiR-210 mitigates OGD/R-induced cardiomyocyte apoptosis

**Fig. 4.**
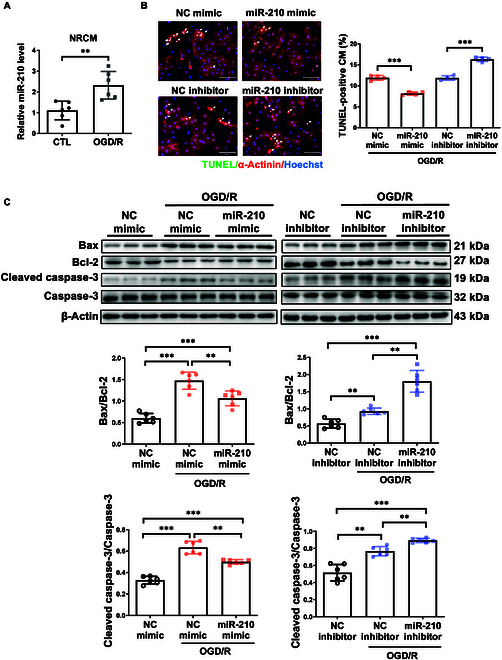
MiR-210 protects cardiomyocytes against OGD/R-induced apoptosis in vitro. (A) qRT-PCR of miR-210 expression in neonate cardiomyocytes stressed with oxygen glucose deprivation/reperfusion (OGD/R) (*n* = 6). (B) TUNEL labeling in miR-210 mimic or inhibitor-transfected NRCMs under OGD/R stress (*n* = 4). Scale bar, 100 μm. (C) Western blot for Bax, Bcl-2, and caspase-3 in miR-210 mimic or inhibitor-transfected NRCM upon OGD/R stress (*n* = 6). For statistical analysis, unpaired Student’s *t* test was performed for (A) and (B). One-way ANOVA test was performed for (C). Data are mean ± SD. **P < 0.01; ***P < 0.001.

Given that molecules regulated by exercise may exert myocardial protection effect, we continued to determine whether miR-210 could regulate apoptosis in neonate cardiomyocytes under the stress of oxygen glucose deprivation/reperfusion (OGD/R). It has been reported that miR-210 responses to hypoxic stress and our data also showed that OGD/R stress induced miR-210 expression in both primary NRCM (Fig. 4A) and AC16 cell line (Fig. S1D). Immunofluorescent staining for TUNEL (terminal deoxynucleotidyl transferase–mediated deoxyuridine triphosphate nick end labeling)/α-actinin and Western blot analysis further revealed that miR-210 overexpression attenuated OGD/R-induced cardiomyocyte apoptosis, while miR-210 inhibition enhanced apoptosis (Fig. 4B and C and Fig. S1E). Thus, miR-210 mitigates apoptosis in cardiomyocytes under OGD/R condition.

### CDK10 is targeted by miR-210 involved in cardiomyocyte proliferation

**Fig. 5.**
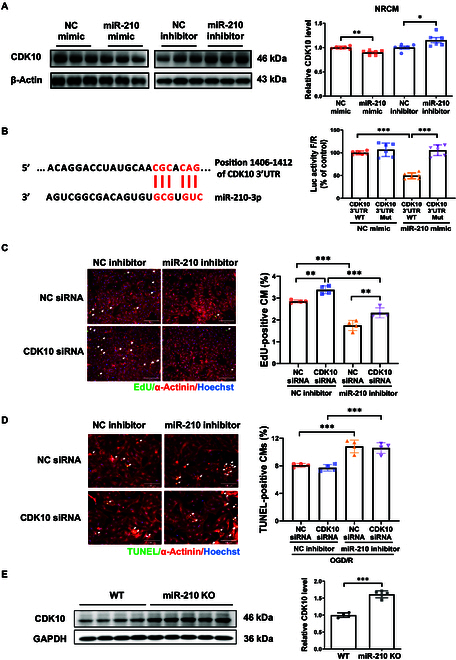
MiR-210 regulates proliferation of cardiomyocytes by targeting CDK10. (A) Western blot for CDK10 in NRCMs with miR-210 overexpression or inhibition (*n* = 6). (B) Luciferase reporter assay to evaluate the direct binding of miR-210 with the 3′UTR of CDK10 (*n* = 6). (C) EdU/α-actinin staining in neonate cardiomyocytes transfected with miR-210 inhibitor and/or CDK10 siRNA (*n* = 4). Scale bar, 200 μm. (D) TUNEL/α-actinin staining in neonate cardiomyocytes transfected with miR-210 inhibitor and/or CDK10 siRNA under OGD/R stress (*n* = 4). Scale bar, 100 μm. (E) Western blot for CDK10 in the rat hearts (*n* = 4 to 5). For statistical analysis, unpaired Student’s *t* test was performed for (A) and (E). Two-way ANOVA test followed by Tukey post hoc test was performed for (B) to (D). Data are mean ± SD. *P < 0.05; **P < 0.01; ***P < 0.001.

To further investigate the downstream targets of miR-210, we performed bioinformatic analysis using miRTarBase and miRWalk, and screened potential target genes that play functional roles in cell proliferation and survival. This included cyclin-dependent kinase 10 (CDK10) and ephrin-A3 (EFNA3). Algorithm analysis predicted CDK10, a cell proliferation regulator, as a downstream target of miR-210 [29,30]; however, its involvement in miR-210’s regulatory effect in cardiomyocytes remained largely unknown. Here, we demonstrated that overexpressing miR-210 down-regulated CDK10, whereas inhibiting miR-210 up-regulated CDK10 (Fig. 5A). The negative regulation of miR-210 on CDK10 expression was similarly found in AC16 cell line (Fig. S2A and C). Meanwhile, luciferase reporter assay indicated that transfection of the plasmids with the 3′ untranslated region (UTR) of CDK10 (predicted to bind with miR-210) together with miR-210 mimic could significantly reduce luciferase activity, while this was not observed after 3′UTR mutation (Fig. 5B), implying a direct interaction between miR-210 and CDK10. Further investigation was conducted through function-rescue experiments in NRCM to ascertain whether CDK10 was involved in miR-210-induced cardioprotection. As determined by EdU labeling, our data demonstrated that miR-210 inhibitor significantly reduced cardiomyocyte proliferation, which was, however, attenuated in NRCM cotransfected with CDK10 small interfering RNA (siRNA) (Fig. 5C). Notably, knockdown of CDK10 did not affect OGD/R-induced cardiomyocyte apoptosis regardless of miR-210 inhibition (Fig. 5D). Finally, we found that CDK10 was up-regulated in heart tissues from miR-210 KO rats (Fig. 5E). Thus, miR-210 also negatively regulated CDK10 in vivo. These findings suggest that CDK10 is targeted by miR-210 in regulating cardiomyocyte proliferation, but it does not regulate cardiomyocyte apoptosis.

### MiR-210 targets EFNA3 regulating both cardiomyocyte proliferation and apoptosis

**Fig. 6.**
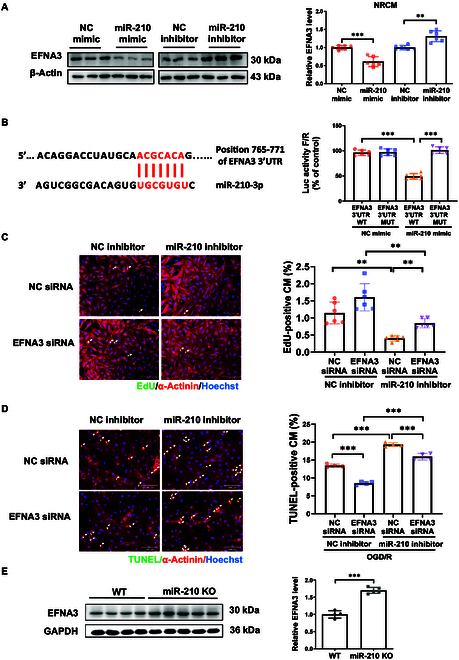
EFNA3 mediates the effect of miR-210 on cardiomyocyte proliferation and apoptosis. (A) Western blot for EFNA3 in NRCMs with miR-210 overexpression or inhibition (*n* = 6). (B) Luciferase reporter assay to evaluate the direct binding of miR-210 with the 3′UTR of EFNA3 (*n* = 6). (C) EdU/α-actinin staining in neonate cardiomyocytes transfected with miR-210 inhibitor and/or EFNA3 siRNA (*n* = 6). Scale bar, 100 μm. (D) TUNEL/α-actinin staining in neonate cardiomyocytes transfected with miR-210 inhibitor and/or EFNA3 siRNA under OGD/R stress (*n* = 4). Scale bar, 100 μm. (E) Western blot for EFNA3 in the rat hearts (*n* = 4 to 5). For statistical analysis, unpaired Student’s *t* test was performed for (A) and (E). Two-way ANOVA test followed by Tukey post hoc test was performed for (B) and (D). Robust two-way ANOVA followed by post hoc pairwiseMedianTest was performed for (C). Data are mean ± SD. **P < 0.01; ***P < 0.001.

Previous studies have reported that EFNA3 is targeted by miR-210 in multiple cell types [31,32]. However, the question of whether miR-210 could regulate EFNA3 in cardiomyocyte proliferation and/or apoptosis remained unanswered. We initially determined EFNA3 expression in primary NRCM and AC16 cell line with miR-210 overexpression or inhibition. We demonstrated that EFNA3 was also negatively regulated by miR-210 at the level of cardiomyocytes (Fig. 6A and Fig. S2B and D). Luciferase reporter assay showed that the 3′UTR sequence of EFNA3 was directly targeted by miR-210 (Fig. 6B). We then performed function-rescue experiments by transfecting NRCM with miR-210 inhibitor and/or EFNA3 siRNA. We found that knockdown of EFNA3 attenuated the proliferation-reducing effect of miR-210 inhibitor (Fig. 6C). Knockdown of EFNA3 also attenuated the effect of miR-210 inhibitor that aggravated cardiomyocyte apoptosis under OGD/R stress (Fig. 6D). Additionally, EFNA3 was detected as being up-regulated in the heart tissues of miR-210 deficiency rats in vivo (Fig. 6E). Our findings, thus, demonstrate that miR-210 targets EFNA3 through which regulates both cardiomyocyte proliferation and apoptosis.

### MiR-210 promotes proliferation and survival of hESC-CMs

**Fig. 7.**
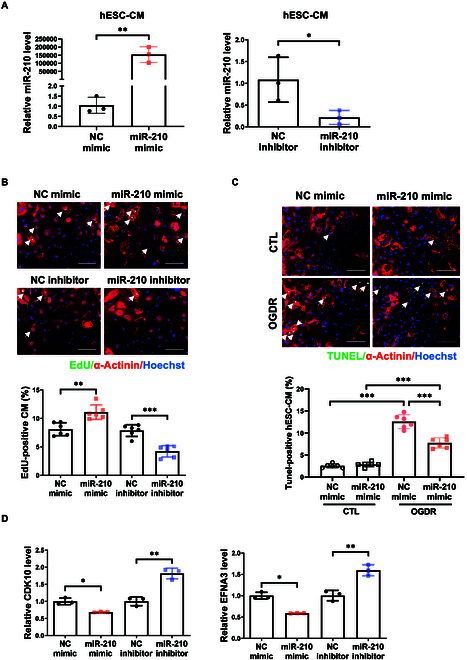
MiR-210 functions in human embryonic stem cell-derived cardiomyocytes. (A) qRT-PCR of miR-210 expression in human embryonic stem cell-derived cardiomyocytes (hESC-CMs) with miR-210 overexpression or inhibition (*n* = 3). (B) EdU/α-actinin immunofluorescent staining in hESC-CM with transfection of miR-210 mimic or inhibitor (*n* = 6). Scale bar, 100 μm. (C) TUNEL staining in miR-210 mimic-transfected hESC-CM under OGD/R stress (*n* = 6). Scale bar, 100 μm. (D) qRT-PCR of CDK10 and EFNA3 expressions in hESC-CM with transfection of miR-210 mimic or inhibitor (*n* = 3). For statistical analysis, unpaired Student’s *t* test was performed for (A), (B), and (D). Two-way ANOVA test followed by Tukey post hoc test was performed for (C). Data are mean ± SD. *P < 0.05; **P < 0.01; ***P < 0.001.

The functional role of miR-210 was further examined in hESC-CM through transfection with mimic or inhibitor targeting miR-210 (Fig. 7A). Functionally, miR-210 mimic markedly increased proliferation; conversely, miR-210 inhibitor reduced proliferation in hESC-CM (Fig. 7B). Increasing miR-210 also inhibited OGD/R-induced apoptosis of human cardiomyocytes (Fig. 7C). We further demonstrated that CDK10 and EFNA3 were negatively regulated by miR-210 in hESC-CM (Fig. 7D). Thus, miR-210 is sufficient to promote the proliferation and survival of human cardiomyocytes, underpinning its potential to be used as an interventional target for clinical treatment.

### MiR-210 mediates exercise-induced cardiac protection against I/R injury

**Fig. 8.**
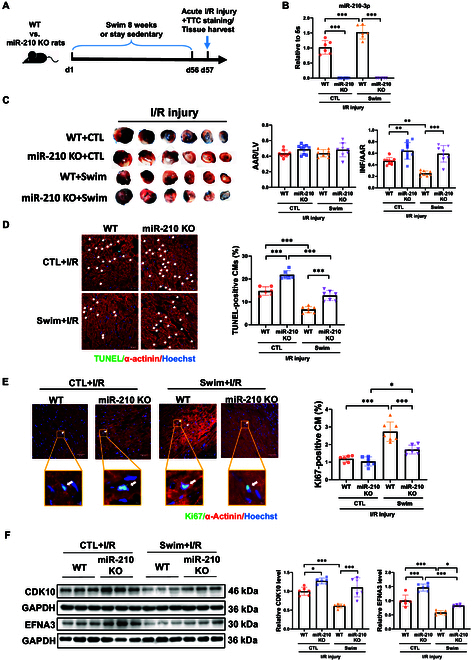
MiR-210 is essential to mediate exercise-driven cardiac protection against I/R injury. (A) Schematic of rat exercise model followed by ischemia/reperfusion (I/R) injury illustrating that adult WT and miR-210 KO rats received swimming exercise or stayed sedentary for 8 weeks, and then were subjected to I/R surgery. (B) qRT-PCR of cardiac miR-210 expression in the swimming and control (CTL) group after I/R surgery (*n* = 6). (C) 2,3,5-Triphenyltetrazolium chloride (TTC) staining of rat hearts showing the area at risk (AAR)/left ventricle weight (LV) ratio and the infarct size (INF)/AAR ratio (*n* = 8 to 10). (D) TUNEL staining in cardiac tissues after I/R injury (*n* = 6 to 7). Scale bar, 50 μm. (E) Immunofluorescent staining for Ki67/α-actinin for analyzing cardiomyocyte proliferative activity (*n* = 6 to 7). Scale bar, 50 μm. An enlarged area was shown below. Scale bar, 5 μm. (F) Western blot for CDK10 and EFNA3 in the rat hearts (*n* = 6). For statistical analysis, two-way ANOVA test followed by Tukey post hoc test was performed for (B) to (F). Data are mean ± SD. *P < 0.05; **P < 0.01; ***P < 0.001.

Due to the results of miR-210 as a significant mediator of exercise-induced cardiomyocyte proliferation and its anti-apoptotic effects in cardiomyocytes, we decided to elucidate its involvement in exercise-driven cardiac protection. We subjected miR-210 KO and WT rats to 8-week swimming exercise, followed by I/R surgery, with the conceptual experimental model shown in Fig. 8A. Our data demonstrated that in I/R WT rats, swimming exercise induced a substantial miR-210 expression in the heart; however, miR-210 KO rats did not exhibit the same miR-210 up-regulation after exercise (Fig. 8B). Notably, exercise significantly decreased the infarction area in WT rats compared to sedentary controls, while miR-210 deficiency resulted in a loss of this exercise-induced protective effect (Fig. 8C). TUNEL staining further indicated that swimming exercise reduced myocardial apoptosis in WT rats upon I/R injury, while this effect was attenuated in miR-210 KO rats (Fig. 8D). Additionally, swimming exercise led to an increase in Ki67-positive cardiomyocytes in WT rats after I/R injury, while miR-210 deficiency significantly abolished the exercise-induced cardiomyocyte proliferation (Fig. 8E). Meanwhile, we observed a significant decrease in both CDK10 and EFNA3, the target genes of miR-210, at the protein level in the exercised group versus sedentary group in WT rats; this reduction was reversed by miR-210 deficiency (Fig. 8F). Collectively, our findings demonstrate that the absence of miR-210 hampers exercise-driven protection against I/R injury in vivo*.* This, in turn, indicates that the presence and exercise-responsive up-regulation of miR-210 are crucial for mediating exercise-induced protection to alleviate I/R injury.

## Discussion

Cardiac I/R injury, a secondary injury after the treatment of myocardial infarction, has attracted considerable attention. Despite this, there are few effective strategies in place to mitigate I/R injury [33]. Accumulating evidence indicates that the mechanisms underpinning exercise-induced cardiac protection and tissue repair provide a new way that might treat I/R injury [34–37]. This study demonstrates that miR-210, an exercise-responsive miRNA, governs exercise-induced cardiac growth and medicates exercise’s effect, which alleviates cardiac I/R injury. Specifically, miR-210 functions to enhance proliferation and reduce apoptosis of cardiomyocytes through its target genes CDK10 and EFNA3. In patients diagnosed of coronary heart diseases, circulating miR-210 can be induced after a programmed cardiac rehabilitation. Increasing miR-210 is also effective to promote proliferation and survival of hESC-CM. These findings suggest its translational value to clinical treatment and indicate that exercise, by inducing miR-210, can effectively mitigate cardiac I/R injury.

Cardiomyocyte death commonly occurs during myocardial injury and represents a significant driver of ventricular remodeling and heart failure [38]. Enhancing cardiomyocyte survival and proliferation is believed to be key to myocardial protection and repair [39]. However, the proliferation capacity of cardiomyocytes is very limited in the adult mammalian heart, constraining the heart’s self-renewal ability after injury [40]. Scientists have used apical resection model to study the cellular and molecular mechanisms of cardiomyocyte proliferation [40,41]. Molecules that are required for cardiac regeneration after apical resection in neonates were found to be able to promote cardiomyocyte proliferation and myocardial repair even in the adult heart [42,43]. Interestingly, previous studies have shown that exercise can induce new cardiomyocyte generation in both healthy and injured adult mouse hearts [44]. In addition to the different molecules (miR-222, miR-17-3p, lncRNA CPhar, Mettl14, etc.) that are known to be involved in this physiological process [15,16,45,46], our study demonstrates that exercise-induced miR-210 is a pivotal molecular mechanism for the exercise-induced proliferative activity of cardiomyocytes, even though it is not necessary for exercise-induced hypertrophy of cardiomyocytes. Consistently, our in vitro experiments show that miR-210 enhances cardiomyocyte proliferation and exerts apoptosis-reducing effect, without influencing cardiomyocyte size. Our data further support that exercise-induced miR-210 is required to mediate exercise’s protection against cardiac I/R injury, which is at least partially attributed to increased proliferative activity and reduced apoptosis of cardiomyocytes. MiR-210 may be a key miRNA involved in the potential contribution of endogenous cardiomyocyte generation to exercise-induced cardiac protection [39].

MiR-210, a hypoxia-responsive microRNA, can be induced with the development of ischemic cardiac diseases [47]. MiR-210 levels also increase in the blood circulation of heart failure patients, suggesting its potential as a predictive biomarker for heart failure or cardiovascular death [48,49]. However, the expression changes and biological functions of peripheral blood and intracellular miRNAs should be analyzed differently. Actually, intracellular miR-210 exhibits protective roles in alleviating cardiac injury and dysfunction, including I/R injury [50]. In addition to strategies that increase miR-210, such as utilizing extracellular vesicles to load and deliver miR-210 [51], exercise has been shown to boost the expression of cardiac miR-210, thereby enhancing angiogenesis in healthy rat hearts [26]. In the present study, we demonstrate that exercise can lead to markedly increased miR-210 expressions in the experimental murine models of swimming exercise. Meanwhile, serum levels of miR-210 were sustainably increased even at 8 days after the end of swimming program in both the sham and cardiac I/R mice. In this case, we would also like to determine whether miR-210 could be induced in healthy individuals or patients after exercise. In our previously reported paper, circulating miR-210 expression levels were not significantly elevated in healthy individuals after a 3-month basketball training [52]. However, to our great notice, circulating miR-210 was markedly induced in those patients diagnosed of coronary heart diseases after an 8-week programmed cardiac rehabilitation. The different response of circulating miR-210 after a 3-month basketball training compared to an 8-week programmed cardiac rehabilitation can be probably related to exercise type (aerobic versus mixed exercise), intensity [targeted intensity set as the heart rate recorded 1 min before attaining the anaerobic ventilation threshold during the cardiopulmonary exercise test (CPET) test versus vigorous intensity], duration (8 weeks versus 3 weeks), and frequency (a 30-session and 3 times a week versus an average of 565 min a week containing amateur basketball matches and other regular training) [28,52]. Our results at least provide evidence that a programmed exercise rehabilitation, which is commonly used as an effective and economic treatment for patients with cardiovascular diseases, can induce miR-210 expression level in the blood circulation.

The functional studies using hESC-CM and human AC16 cells further show that miR-210 is effective to promote proliferation and survival of human cardiomyocytes. Thus, miR-210, an exercise-responsive miRNA, has obvious protective effects for cardiomyocytes, including human cardiomyocytes. Furthermore, the animal experimental I/R models were implemented in miR-210 KO rats with or without swimming exercise, showing that exercise can also effectively induce miR-210 expression in the injured myocardium, thus providing protection for the heart. It is intriguing that both physiological stimulus (e.g., exercise training) and pathological stress (e.g., cardiac I/R injury) can induce miR-210 expression in the heart. Accumulating evidence has supported that miR-210 is increasingly expressed under hypoxic conditions through the hypoxia-inducible factor (HIF) pathway [53]. Interestingly, miR-210 can also be activated in HIF-independent pathway [21]. We hypothesize that the AKT activation may be instrumental in the induction of miR-210 following exercise training [9,54]. Further investigations applying RNA-sequencing and proteomics of myocardium after physiological exercise and/or pathological ischemic and hypoxic stress will be useful to elucidate the upstream regulators of miR-210 in these different conditions. Herein, we propose that exercise, a nondrug intervention, can serve as a potent tool to stimulate the endogenous expression of miR-210 in heart, thereby exerting cardioprotective effects. The underlying mechanism of increased miR-210 in response to exercise and the influence of different exercise type and regimen on cardiac miR-210 expression deserve further investigation.

Based on the observations of miR-210-regulated cardiomyocyte proliferation and apoptosis in I/R injury, we sought to screen potential downstream targets of miR-210. Our focus fell on CDK10 and EFNA3. CDK10, an essential regulator of the cell cycle, is essentially involved in regulating cell proliferation. Previous studies have highlighted the dual roles of CDK10 that can function as tumor suppressor or oncogene in cancers [55–57]. Lower level of CDK10 was associated with resistance to endocrine therapy in breast cancer patients [58]. The methylation of CDK10 promoter or ubiquitination of CDK10 has been shown among the mechanisms of CDK10 regulation in cancers [58,59]. However, its role in the myocardium has been relatively underexplored. In addition, we evaluated EFNA3, a previously identified target of miR-210 [31,32], in the myocardium and in the I/R injury model after swimming regimen. Increasing evidence has indicated that EFNA3 participates to regulate apoptosis in different cell types such as nucleus pulposus cells, vascular endothelial cells, Müller cells, and sensory axon [59–62]. Lower EFNA3 expression was previously demonstrated in the peri-infarct myocardium with miR-210 overexpression treatment [23]; however, the cell type-specific study from which the exact contribution of EFNA3 to regulate cardiomyocyte proliferation or survival was unclear. Here, we demonstrate that miR-210 can directly target CDK10 and EFNA3, and show that miR-210 efficiently down-regulates CDK10 and EFNA3 expressions in primary NRCM, AC16 cardiomyocyte cell line, and hESC-CM. Meanwhile, we found that in the I/R hearts, exercise could up-regulate miR-210 while simultaneously down-regulating CDK10 and EFNA3 expressions. However, these changes were attenuated in miR-210 KO rats. These results unveil a novel regulatory mechanism of miR-210, where CDK10 and EFNA3 are revealed as target genes of miR-210, which regulate cardiomyocyte proliferation. Additionally, EFNA3 down-regulation also mediates the apoptosis-reducing effect of miR-210 in cardiomyocytes. These results shed new light on the mechanisms by which miR-210 contributes to exercise-driven protection upon the I/R injury.

Noteworthy, in addition to CDK10 and EFNA3 that we have demonstrated to mediate the functional role of miR-210 in promoting cardiomyocyte proliferation and/or inhibiting apoptosis, other potential downstream target genes of miR-210 might regulate cardiomyocyte functions and be involved in the protective effect of miR-210 against cardiac I/R injury. Moreover, in addition to cardiomyocytes, miR-210 has also been reported to be altered in endothelial cells, which can regulate angiogenesis and participate in cell cross talk through paracrine actions in ischemic heart diseases [53]. As here we used miR-210 KO rats in the exercised model and cardiac I/R experiment, it should be taken into consideration that other types of cells (in addition to cardiomyocytes) might also take effect to mediate the protections of exercise against cardiac I/R injury, which deserve further investigations.

In conclusion, we elucidate the role of miR-210, an exercise-responsive miRNA, in promoting the proliferative activity of cardiomyocytes during physiological cardiac growth when induced in an exercised heart. MiR-210 enhances proliferation and exerts apoptosis-reducing effect by targeting CDK10 and EFNA3. Furthermore, miR-210 essentially contributes to exercise-driven protection against cardiac I/R injury. MiR-210 also functions in human cardiomyocytes and can be induced in patients with coronary heart diseases after programmed cardiac rehabilitation. These findings provide robust evidence for the underlying mechanism of exercise as an effective nondrug intervention. By inducing miR-210, exercise promotes the survival and proliferative activity of cardiomyocytes and alleviates I/R injury in the heart.

## Methods

Patients diagnosed of coronary heart diseases were recruited in Shanghai Xuhui Central Hospital (Shanghai, China) for blood sample collection, with the protocol approved by the Ethics Committee of Shanghai Xuhui Central Hospital (number 2016-10) and the written informed consents given by the participants before enrollment. All animal experiments were approved by the Shanghai University Committee for the Ethics of Animal Experiments. The animal experiments were performed under the Guidelines concerning laboratory animals for biomedical research published by the National Institutes of Health (no. 85-23, revised 1996).

### Participants and cardiac rehabilitation program

From June 2016 to December 2018, totally 20 patients diagnosed of coronary heart diseases were recruited in Shanghai Xuhui Central Hospital [63]. The coronary heart disease was diagnosed by a cardiologist according to the clinical symptoms and medical examinations by electrocardiogram, echocardiography, and/or coronary angiogram. Patients took CPET before the cardiac rehabilitation program. Patients underwent targeted intensity cardiac rehabilitation (3 times per week, 8-week duration), consisting of 5-min warm up, 20-min cycle ergometer aerobic exercise training with targeted intensity (ranging from 70% to 80% of the estimated heart rate peak and recorded 1 min before attaining the anaerobic ventilation threshold during the test), and 5-min slow down as previously reported [28]. The human serum samples were collected before the cardiac rehabilitation program and right after the last exercise training and then stored at −80 °C until determination of circulating miR-210 levels.

### Generation of miR-210 KO rat ESC and rat animal

A total of 12 μg of homologous targeting plasmids was transferred into 5 × 10^5^ rat ESCs using the electroporation method to achieve miR-210 KO in rat ESC. Then, the cells were plated into G418-resistant feeder-coated 35-mm dishes and further selected by G418 (GIBCO, 200 μg/ml) for 7 days to obtain single colony, which was amplified and genotyped using polymerase chain reaction (PCR). The primer used (forward and reverse) was as follows: ctgttcctgcctctaatcaaggttatag and caccttggagccgtactggaac. MiR-210 KO rats were generated by diploid blastocyst injection and mutual breeding and mating. The miR-210 KO rats were provided by W. Li (State Key Laboratory of Stem Cell and Reproductive Biology, Institute of Zoology, Chinese Academy of Sciences).

### Animal model and swimming exercise regimen

Eight-week-old male miR-210 KO and wild-type (WT) rats were maintained in specific pathogen-free (SPF) animal facility (Shanghai University, Shanghai, China). Adult miR-210 KO rats and WT rats received either a swimming exercise for 8 weeks or remained sedentary. Briefly, the swimming exercise regimen began with a 1-week adaptation period of 10-min sessions twice daily (once in the morning and once in the afternoon), escalating 10 min each day until reaching 60 min twice daily [27]. From the third day, an additional 3% body weight (BW) load was introduced. The swimming duration, inclusive of the adaptation period, spanned 8 weeks. After 8 weeks, rats were anesthetized followed by heart tissue harvest. The HW and the HW relative to BW or tibia length (TL) were determined [15,16]. Rat serum samples, heart tissues, or optimal cutting temperature compound (OCT)-embedded heart tissues were frozen and stored at −80 °C. To determine the cardiac and circulating miR-210 levels in exercised mice, male adult C57BL/6J mice purchased from Charles River (Beijing, China) took a 4-week swimming exercise regimen without BW load as previously reported [15,16]. At the end of swimming exercise, mice serum samples were collected. To further investigate whether circulating miR-210 was sustainably increased after exercise, mice were subjected to swimming exercise for 3 weeks and then cardiac I/R injury (ischemia for 30 min and then reperfusion) as reported previously [35]. At 8 days after the end of swimming exercise (7 days after I/R or sham surgery), mice serum samples were collected to measure circulating miR-210 level.

### Cardiac I/R injury

The involvement of miR-210 in exercise’s protection was studied by using the miR-210 deficiency rats or WT controls that either received swimming exercise or remained sedentary; following 8 weeks of swimming exercise or sedentary living, a cardiac I/R injury model was implemented [35,64]. After anesthetization and endotracheal ventilation for rats, the I/R surgery (ligation, 1 h; reperfusion, 3 h) was performed at the level of left anterior descending coronary artery [35]. It has been reported that from 2 to 3 h after reperfusion, the injured heart can develop obvious myocardial injury, including increased infarct size, cardiac myocyte death, oxidative stress and mitochondrial metabolic dysfunction, and increased circulating level of lactate dehydrogenase (LDH) [65–68]. Infarct size was determined by 2,3,5-triphenyltetrazolium chloride staining [35]. Subsequently, heart tissues or OCT-embedded heart tissues were frozen and stored at −80 °C.

### Wheat germ agglutinin staining

Cardiac cryosections (5 μm thick) were treated with 4% polyformaldehyde (PFA) for 20 min. Following phosphate-buffered saline (PBS) washes, wheat germ agglutinin (WGA; Sigma L4895a) staining was performed [16]. The myocardium’s cross-sectional area was observed under confocal microscopy (Carl Zeiss, LSM710) and analyzed with ImageJ software.

### Cardiomyocyte culture and transfections

Sprague-Dawley (SD) rats at postnatal days 1 to 3 were used to isolate neonate cardiomyocytes. Neonate rat hearts were minced and digested with pancreatin (Sigma, P3292) and collagenase II (Gibco, 17101015) and then treated with Percoll (GE Healthcare, 17-0891-01) centrifugation [15]. Neonate cardiomyocytes were cultured with high-glucose Dulbecco’s modified Eagle’s medium (DMEM; Corning), which contained 5% fetal bovine serum (FBS; BioInd, Israel) and 10% horse serum (Gibco). To investigate the functions of miR-210 in cardiomyocytes, transfections of 50 nM miR-210 mimic or 100 nM miR-210 inhibitor (RiboBio, Guangzhou, China) were conducted for 48 h using Lipofectamine 2000 (Invitrogen). The cotransfection of miR-210 inhibitor (100 nM) and siRNA targeting CDK10 or EFNA3 (75 nM, RiboBio, Guangzhou, China) was conducted in NRCM to investigate the involvement of CDK10 and EFNA3 in miR-210-regulated cardiomyocyte proliferation and apoptosis. Meanwhile, the functions of miR-210 were investigated in hESC-CMs and in human AC16 cardiomyocyte cell line as well. The hESCs were cultured and differentiated to cardiomyocytes as previously reported [61]. AC16 cells were maintained in DMEM (10% FBS). Both hESC-CM and AC16 cells were transfected with miR-210 mimic or inhibitor as described above. At 48 h after transfection, cells were harvested for analysis.

### OGD/R stress

The OGD/R stress was implemented in cardiomyocytes serving as a widely used cellular model to mimic cardiac I/R injury. Briefly, NRCM or AC16 cells were exposed to a hypoxic condition (<1% O_2_) in glucose- and serum-deprived DMEM for 8 h. Following this, cells were cultured in DMEM with serum and glucose under normoxic conditions for 12 h as previously reported [35]. Transfections with miR-210 mimic/inhibitor, CDK10/EFNA3 siRNA, or negative controls were performed for 48 h as previously described before the end of OGD/R stress. After 20 h of OGD/R stress, TUNEL staining was conducted in NRCM or AC16 cells. For hESC-CM, cells were subjected to OGD/R stress (oxygen glucose deprivation, 16 h; reperfusion, 12 h) for a total of 28 h before cell harvest.

### Ki67 and EdU stainings

Cardiac cryosections (5 μm thick) or cultured cardiomyocytes were examined with immunofluorescent stainings for α-actinin (diluted with 1:200, Sigma, A7811) and Ki67 (diluted with 1:100, Abcam, ab16667) or stained with α-actinin (diluted with 1:200, Sigma, A7811) and Cell-Light EdU Apollo488 In Vitro Kit (KeyGEN, KGA331) for EdU labeling as previously reported [41]. The ratio of Ki67-positive or EdU-positive cardiomyocytes was observed under confocal microscopy (Carl Zeiss, LSM710) or fluorescence microscopy (Leica, DMi8) and analyzed using ImageJ software.

### TUNEL staining

Cardiac cryosections (5 μm thick) or cardiomyocytes were examined for cardiomyocyte apoptosis using TUNEL FITC Apoptosis Detection Kit (Vazyme, A111-03) [35]. α-Actinin staining was performed to label the cardiomyocytes. Images were taken with confocal microscopy (Carl Zeiss, LSM710) and analyzed for TUNEL-positive cardiomyocytes using ImageJ software.

### Luciferase reporter assay

To evaluate the direct interaction between miR-210 and its target genes, the binding sequences (mutated or not) in the 3′UTR of CDK10 and EFNA3 were inserted into the PGL3-Basic luciferase reporter vector. The miR-210 mimic and the luciferase reporter-inclusive plasmids were cotransfected into human embryonic kidney (HEK) 293 cells. Dual-Luciferase Reporter Assay (Promega) was used for luciferase activity measurement of Firefly and Renilla. The sequences of CDK10 and EFNA3 binding sites used for luciferase reporter assay are listed in Table S1.

### Western blot

Cardiac tissues or cardiomyocytes were lysed with radioimmunoprecipitation assay (RIPA) lysis buffer (Beyotime, China) plus protease and phosphatase inhibitor (Beyotime, China). Western blot was performed with specific primary antibodies: Bcl-2 (Abclonal, A2845), Bax (Abclonal, A0207), caspase-3 (Abclonal, A19654), CDK10 (Abclonal, A2690), or EFNA3 (Abclonal, A2724 or Proteintech, 12480-1-AP). β-Actin or glyceraldehyde-3-phosphate dehydrogenase (GAPDH) was used for internal controls.

### Quantitative reverse transcription polymerase chain reaction

Total RNA was extracted from cardiac tissues or cardiomyocytes. The Trizol RNAiso Plus kit (TaKaRa, 9109) was used for RNA extraction, and the RevertAid First Strand cDNA Synthesis Kit (Thermo Fisher Scientific, K1622) was used for reverse transcription (RT). The quantitative PCR was then performed using the following primers (5′-3′): rno-ANP-F: GAGCAAATCCCGTATACAGTGC; rno-ANP-R: ATCTTCTACCGGCATCTTCTCC; rno-BNP-F: CTGCTTGCGGAGGCGAGAC; rno-BNP-R: TGTTCTGGAGACTGGCTAGGACTTC; hsa-CDK10-F: GCCTGCGTCATCCGAACAT; hsa-CDK10-R: AGGGTGTTGGCATATTCTCCA; hsa-EFNA3-F: CATGCGGTGTACTGGAACAG; hsa-EFNA3-R: AGATAGTCGTTCACGTTCACCT. The miR-210 expression in heart tissues or cardiomyocytes was determined by qRT-PCR (RiboBio, Guangzhou, China). 18*S* or 5*S* was used for internal control as appropriate. For analysis of circulating miR-210 levels, total RNA was extracted from serum samples using MirVana miRNA isolation kit (Thermo Fisher Scientific, AM1561). *Caenorhabditis elegans* miR-39 (cel-miR-39) was spiked in to normalize the blood volume. The primers for miRNA detection in the blood sample by PCR were obtained from RiboBio (Guangzhou, China).

### Statistical analysis

All statistical analyses were performed using SPSS 20.0 or GraphPad Prism 8. Data were presented as mean ± SD. All data were first analyzed by normality distribution test. For the data that passed normality test, unpaired Student’s *t* test was used for statistical analyses between 2 groups; one-way analysis of variance (ANOVA) test was used for statistical analyses among 3 groups; two-way ANOVA test and Tukey post hoc test were applied for comparisons among 4 groups. For the data that did not pass normality test, we used Mann–Whitney *U* test to compare differences between 2 groups, and used robust two-way ANOVA followed by post hoc pairwiseMedianTest in the rcompanion package for comparisons among multiple groups. Paired Student’s *t* test was used to compare the difference of human serum miR-210 expression levels before and after the cardiac rehabilitation program. *P* < 0.05 indicates statistical significance.

## Data Availability

The data reported are available in the article itself or in its online Supplementary Materials.
