# Supplementary material for: Exercise-Induced miR-210 Promotes Cardiomyocyte Proliferation and Survival and Mediates Exercise-Induced Cardiac Protection against Ischemia/Reperfusion Injury
Source: Research (Wash D C). 2024 Feb 26;7:0327. doi: 10.34133/research.0327 (PMC10895486; doi:10.34133/research.0327)
Supplement: Supplementary 1 — Table S1 Figs. S1 and S2 [file research.0327.f1.docx]

**Supplemental Materials**

**Table S1. Sequences for luciferase reporter assays**

|  | **Sequences (5’−3’)** |
| --- | --- |
| CDK10-3’UTR-WT-F | GAGATCATAACTGTTCTTGCTTGGCGACTAGGAGCCGCCCAGGACAGATAAGCTATAGTC |
| CDK10-3’UTR-WT-R | GACTATAGCTTATCTGTCCTGGGCGGCTCCTAGTCGCCAAGCAAGAACAGTTATGATCTC |
| CDK10-3’UTR-MUT-F | GAGATCATAACTGTTCTTGCTTGGCGACTAGGAGCGCGCGTCGACAGATAAGCTATAGTC |
| CDK10-3’UTR-MUT-R | GACTATAGCTTATCTGTCGACGCGCGCTCCTAGTCGCCAAGCAAGAACAGTTATGATCTC |
| EFNA3-3’UTR-WT-F | CCTCTTTTGTCTTCTGTGAAGACAGGACCTATGCAACGCACAGACACTTTTGGAGACCGTAAC |
| EFNA3-3’UTR-WT-R | GTTACGGTCTCCAAAAGTGTCTGTGCGTTGCATAGGTCCTGTCTTCACAGAAGACAAAAGAGG |
| EFNA3-3’UTR-MUT-F | CCTCTTTTGTCTTCTGTGAAGACAGGACCTATGCATGCGTGTGACACTTTTGGAGACCGTAAC |
| EFNA3-3’UTR-MUT-R | GTTACGGTCTCCAAAAGTGTCACACGCATGCATAGGTCCTGTCTTCACAGAAGACAAAAGAGG |

The underlined section is the seed sequences or mutated seed sequences.

**Figure S1**


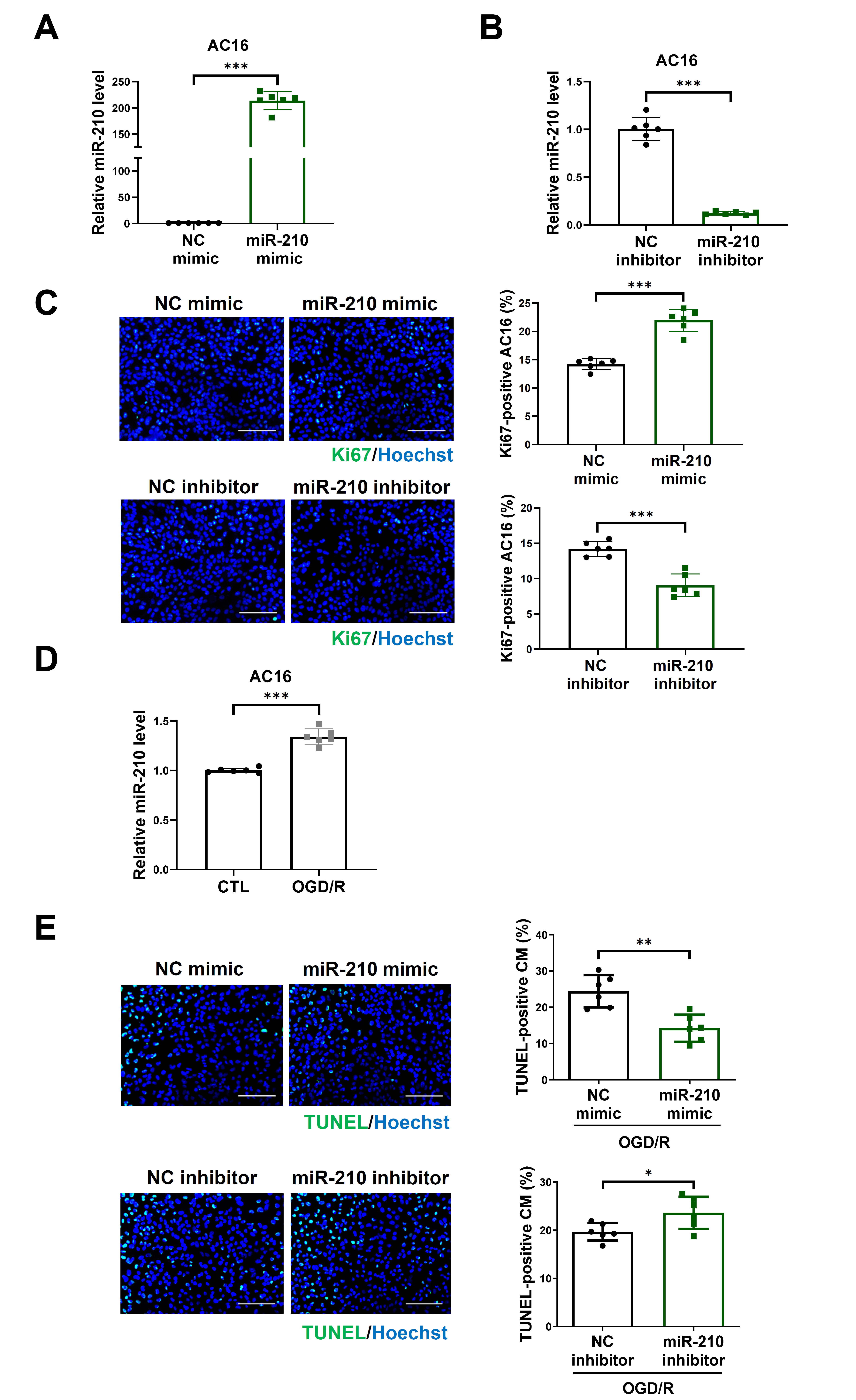


**Figure S1. MiR-210 promotes proliferation and inhibits apoptosis of human AC16 cell line *in vitro*. A and B,** qRT-PCR of miR-210 expression in human AC16 cell line with transfection of miR-210 mimic **(A)**, miR-210 inhibitor **(B)**, or negative controls (NC) (n=6). **C,** Ki67 immunofluorescent staining in AC16 cells with miR-210 overexpression or inhibition (n=6). Scale bar=100 μm. **D,** qRT-PCR of miR-210 expression in AC16 cells stressed with oxygen glucose deprivation/reperfusion (OGD/R) (n=6). **E,** TUNEL staining in miR-210 mimic or inhibitor transfected AC16 cells under OGD/R stress and the ratio of TUNEL-positive cardiomyocytes were recorded (n=6). Scale bar=100 μm. For statistical analysis, unpaired Student’s t-test was performed for **A-E**. Data shown as mean±SD. *, *P*<0.05; **, *P*<0.01; ***, *P*<0.001.

**Figure S2**


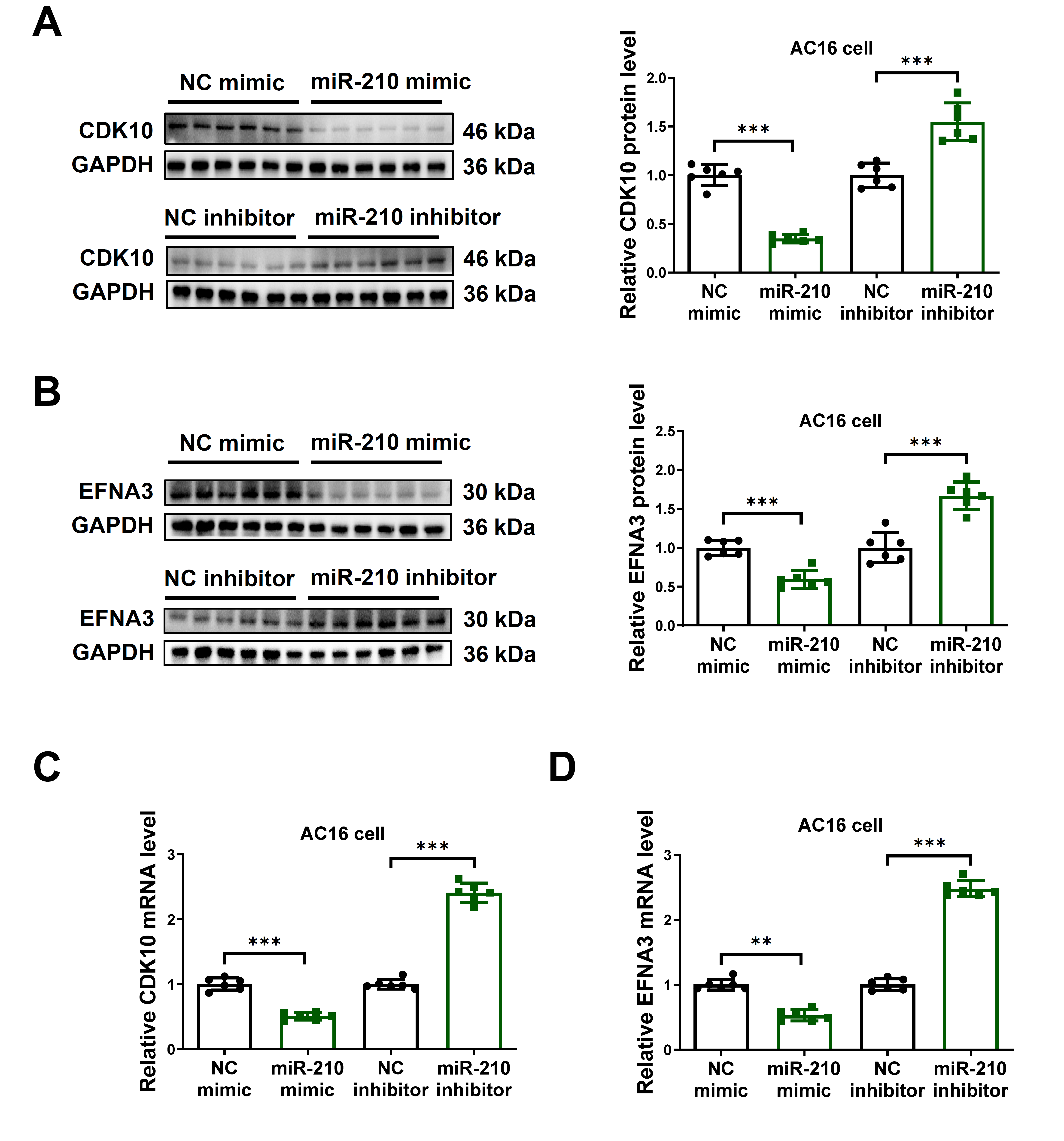


**Figure S2. miR-210 regulates CDK10 and EFNA expressions in human AC16 cell line. A and B,** Western blot for CDK10 **(A)** and EFNA3 **(B)** in AC16 cells with transfection of miR-210 mimic, miR-210 inhibitor, or negative controls (NC) (n=6). **C and D,** qRT-PCR for CDK10 **(C)** and EFNA3 **(D)** in AC16 cells with transfection of miR-210 mimic, miR-210 inhibitor, or negative controls (NC) (n=6). For statistical analysis, unpaired Student’s t-test was performed for **A-C, and D** (EFNA3 expression in AC16 transfected with miR-210 inhibitor). Mann Whitney U test was performed for **D** (EFNA3 expression in AC16 transfected with miR-210 mimic). Data shown as mean±SD. **, *P*<0.01; ***, *P*<0.001.
